# Structure and ion-dependent folding of k-junctions

**DOI:** 10.1261/rna.079678.123

**Published:** 2023-09

**Authors:** Mengxiao Li, Jie Deng, Xuemei Peng, Jia Wang, Timothy J. Wilson, Lin Huang, David M.J. Lilley

**Affiliations:** 1 Guangdong Provincial Key Laboratory of Malignant Tumor Epigenetics and Gene Regulation, Guangdong-Hong Kong Joint Laboratory for RNA Medicine, Sun Yat-Sen Memorial Hospital, Sun Yat-Sen University, Guangzhou 510120, China; 2 College of Pharmacy, Shenzhen Technology University, Shenzhen 518118, China; 3 Nucleic Acid Structure Research Group, MSI/WTB Complex, The University of Dundee, Dundee DD1 5EH, United Kingdom

**Keywords:** RNA structure, metal ions, TPP riboswitch, fluorescence resonance energy transfer, X-ray crystallography

## Abstract

k-Junctions are elaborated forms of kink turns with an additional helix on the nonbulged strand, thus forming a three-way helical junction. Two were originally identified in the structures of *Arabidopsis* and *Escherichia coli* thiamine pyrophosphate (TPP) riboswitches, and another called DUF-3268 was tentatively identified from sequence information. In this work we show that the *Arabidopsis* and *E. coli* riboswitch k-junctions fold in response to the addition of magnesium or sodium ions, and that atomic mutations that should disrupt key hydrogen bonding interactions greatly impair folding. Using X-ray crystallography, we have determined the structure of the DUF-3268 RNA and thus confirmed that it is a k-junction. It also folds upon the addition of metal ions, though requiring a 40-fold lower concentration of either divalent or monovalent ions. The key difference between the DUF-3268 and riboswitch k-junctions is the lack of nucleotides inserted between G1b and A2b in the former. We show that this insertion is primarily responsible for the difference in folding properties. Finally, we show that the DUF-3268 can functionally substitute for the k-junction in the *E. coli* TPP riboswitch such that the chimera can bind the TPP ligand, although less avidly.

## INTRODUCTION

**FIGURE 1.**
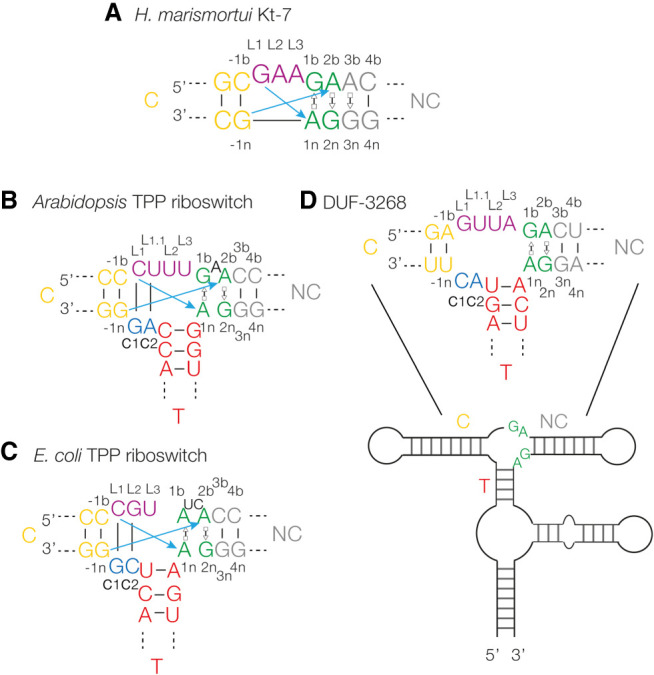
Sequences of riboswitch k-junctions and DUF-3268. (*A*) The sequence of *Haloarcula marismortui* Kt-7, a standard k-turn. The nucleotide positions are colored in our normal scheme and labeled with the normal k-turn nomenclature (Liu and Lilley 2007). Both coloring and nomenclature have been applied to the k-junctions, and used throughout. The blue arrows show the two cross-strand hydrogen bonds from L1 O2′ to A1n N1 and L-1n to A2b N1 or 3 (this varies between k-turns). (*B*,*C*) The sequences of the *Arabidopsis* (*B*) and *Escherichia coli* (*C*) TPP riboswitch k-junctions are shown on the *left*. The main differences from the simple k-turns are the presence of a bulge between G1b and A2b, and the connecting sequence (c1 and c2) and T-helix on the *lower* strand. (*D*) The secondary structure of the DUF-3268 RNA, with the sequence of the putative k-junction.

Although RNA structure determination and analysis techniques have advanced greatly, understanding and predicting the tertiary structure of RNA from the primary and secondary structure is still not fully solved. There are two reasons for this. First, only about 200 unique RNA structures have been solved. Second, too few RNA motifs have been studied in detail. RNA structures can be usefully analyzed as a collection of helical duplex sections connected by junctions that determine the trajectory and mediate long-range interactions. One quite widespread junction is the kink-turn (k-turn); a motif commonly found in duplex RNA comprising a short (usually 3 nt) bulge-loop followed by conserved G:A and A:G sheared base pairs (Fig. 1A; Klein et al. 2001). k-turns can be induced to fold (e.g., by addition of metal ions) into a tightly kinked structure with an included angle of ∼50° (Goody et al. 2004; Liu and Lilley 2007), stabilized by critical cross-strand hydrogen bonds between 2′-hydroxyl groups and ring nitrogen atoms of the adenine nucleobases of the G:A base pairs (Lescoute et al. 2005; Liu and Lilley 2007; Reblova et al. 2011; Daldrop and Lilley 2013).

k-junctions are elaborated forms of k-turns, preserving the tandem G:A base pairs and cross-strand hydrogen bonding, but having an additional third helix on the *n* (nonbulged) strand opposite the loop on the b (bulged) strand (Fig. 1B,C). These were first identified in the structures of the *Arabidopsis* and *E. coli* TPP riboswitches (Serganov et al. 2006; Thore et al. 2006) using a computer algorithm that searched for k-turn features with the correct relative orientation (Wang et al. 2014). In the TPP riboswitches, the k-junctions are not directly involved in ligand binding. Rather, they determine the trajectory of two adjacent helices (termed the C- and NC-helices) that have internal loops that form the TPP binding site (Serganov et al. 2006; Thore et al. 2006). The k-junction is not restricted to riboswitches; two three-way RNA junctions that conform to the k-junction pattern were also identified within the *H. marismortui* ribosome structure (Wang et al. 2014).

The application of bioinformatics has proved to be a powerful approach to identifying potentially functional structured regions of RNA, and the Breaker laboratory has developed comparative genomic analysis to seek out conserved secondary structured regions within intergenic regions (Weinberg et al. 2007; Weinberg et al. 2010; Li and Breaker 2017). Using this approach, Weinberg et al. (2017) identified 224 novel structured RNA sequences. From inspection of their data, we identified four probable k-turn structures, and proved this experimentally in solution and in the crystal (Huang et al. 2021). In addition to these k-turns, we also identified a candidate k-junction (Fig. 1D) in a sequence termed DUF-3268 (Huang and Lilley 2018; Huang et al. 2021).

In this work we have asked two questions. First, do the k-junctions found in the TPP riboswitches undergo folding in solution into the kinked geometry that is observed in the crystal driven by the addition of metal ions? This has not been examined experimentally for any k-junction to date. Second, can we demonstrate that the DUF-3268 sequence adopts the k-junction conformation experimentally? We have determined the crystal structure of this motif and shown that it does indeed adopt the conformation of a k-junction. Furthermore, like the TPP riboswitch k-junctions, folding is promoted by the addition of both divalent and monovalent metal ions in a two-state conformational change.

## RESULTS

### Ion-induced folding of TPP riboswitch k-junctions

**FIGURE 2.**
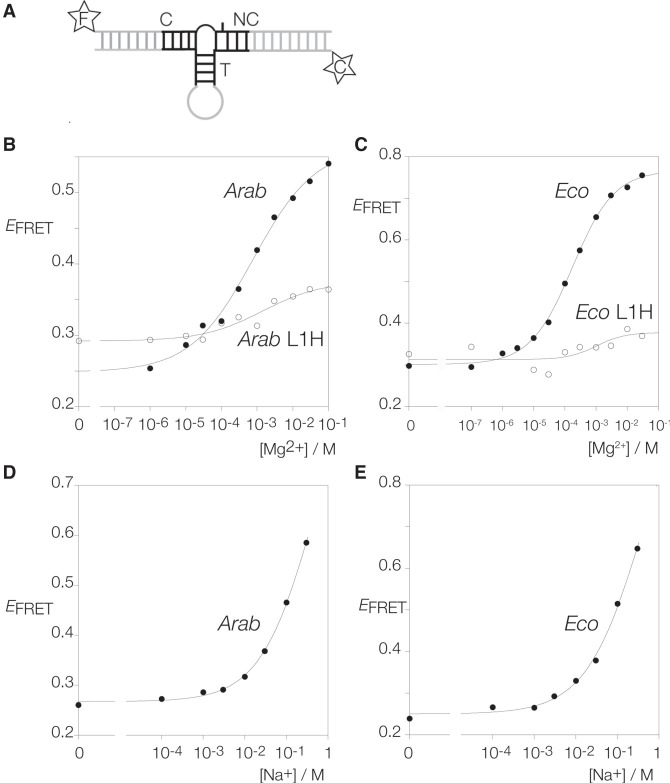
Metal ion-induced folding of the *Arabidopsis* and *E. coli* TPP riboswitch k-junctions analyzed by FRET. (*A*) Schematic of the constructs used for FRET analysis of k-junctions. The T-helix has been replaced by a 4 bp stem–loop, and the C- and NC-helices have been extended and labeled with 5′ fluorescein (F) and cyanine-3 (C), respectively. The *Arabidopsis* and *E. coli* k-junctions have been titrated with metal ions, and FRET efficiency (*E*_FRET_) measured and plotted as a function of ion concentration. The data are fitted to a simple two-state binding model (lines). (*B*) Titration of the *Arabidopsis* k-junction with Mg^2+^ ions. *Filled circles*: natural sequence *Arabidopsis* k-junction. *Open circles*: *Arabidopsis* k-junction with L1H substitution. This removes the hydroxyl group proposed to donate a hydrogen bond to A1n N1. (*C*) Titration of the *E. coli* k-junction with Mg^2+^ ions. *Filled circles*: natural sequence *E. coli* k-junction. *Open circles*: *E. coli* k-junction with L1H substitution. (*D*) Titration of the *Arabidopsis* k-junction with Na^+^ ions. (*E*) Titration of the *E. coli* k-junction with Na^+^ ions.

The clear structural relationship between the TPP riboswitch k-junctions and standard k-turns suggests that it is likely the former will undergo folding into a kinked geometry on the addition of metal ions, yet this has never been shown experimentally. In order to study the anticipated ion-induced folding of a k-junction, we took the *Arabidopsis* and *E. coli* TPP riboswitch junctions and converted them into species similar to those used previously for analysis of the folding of simple k-turns. To achieve this, we extended the C- and NC-helices to 12 and 13 bp, respectively, and converted the T-helices into 4 bp stem–loops (Fig. 2A; Supplemental Fig. S1). Fluorescein and cyanine-3 fluorophores were attached to the 5′ termini of the C- and NC-helices, respectively, in order to study folding by fluorescence resonance energy transfer (FRET). By analogy with standard k-turns, folding would be expected to kink the structure at the junction, so bringing the fluorophores at the helical termini closer together, and thus increase the efficiency of energy transfer (*E*_FRET_). FRET efficiency was measured in the steady-state, using the acceptor normalization method (Clegg 1992).

The addition of Mg^2+^ ions to either k-junction leads to a marked increase in FRET efficiency (Fig. 2B,C), consistent with folding into a more kinked geometry on the addition of the divalent metal ions. The *E*_FRET_ value for the *Arabidopsis* k-junction increases from 0.25 in the absence of ions to 0.57 in the presence of 100 mM Mg^2+^ (Fig. 2B). This is very similar to the change observed for standard k-turns such as *H. marismortui* Kt-7 (Liu and Lilley 2007), suggesting a similar extent of kinking, consistent with the structure of the junction in the riboswitch. The titration was well fitted by a simple two-state binding model, yielding a [Mg^2+^]_1/2_ = 770 µM (Table 1). A somewhat larger change in *E*_FRET_ was induced by the addition of Mg^2+^ ions to the corresponding RNA construct containing the *E. coli* k-junction, with a [Mg^2+^]_1/2_ = 170 µM (Table 1).

**TABLE 1.**
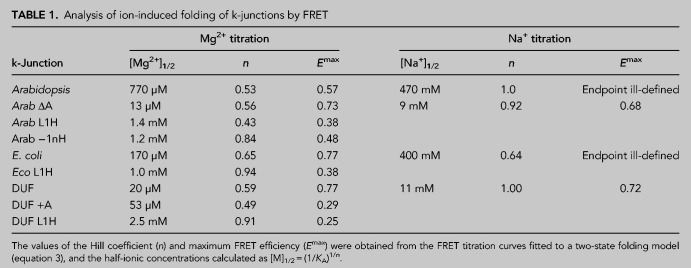
Analysis of ion-induced folding of k-junctions by FRET

Both k-junctions also folded in response to the addition of monovalent metal ions. FRET efficiency increased with the addition of Na^+^ ions (Fig. 2D,E), with [Na^+^]_1/2_ = 470 and 400 mM for the *Arabidopsis* and *E. coli* k-junctions, respectively (Table 1). The endpoints are harder to define with the monovalent ions, but similar folding is apparently achieved, although this requires three orders of magnitude greater concentration for sodium ions, similar to the folding of standard k-turns.

Standard k-turn structures undergo folding upon addition of the L7Ae protein (Turner et al. 2005; Wang et al. 2012; Huang and Lilley 2013). However, on the addition of *Archeoglobus fulgidus* L7Ae protein to the *Arabidopsis* k-junction construct no increase in FRET efficiency was observed. At a concentration of 1 µM L7Ae, *E*_FRET_ = 0.36, that is, no significant increase above the background in buffer alone was detected.

### The effect of atomic mutation on ion-induced folding of TPP riboswitch k-junctions

In a standard k-turn there are two important cross-strand k-turns (Lescoute et al. 2005; Liu and Lilley 2007; Reblova et al. 2011; Daldrop and Lilley 2013). One is donated by the L1 O2′ to A1n N1 (Lescoute et al. 2005; Liu and Lilley 2007), while the other is donated by the O2′ at the −1n position to either N3 or N1 of A2b (Reblova et al. 2011; Daldrop and Lilley 2013). The assignment of these hydrogen bond donors in the k-junction is not obvious from sequence alone. Inspection of the *Arabidopsis* and *E. coli* k-junctions indicated that L1 should be assigned to the nucleotide base paired with the c1 nucleotide (so that the nucleotide 3′ to L1 was termed L1.1, whereupon the following L2 and L3 take their usual functions). The −1b:−1n base pair then followed L1:c1 as the next base pair in the C-helix. Thus the −1n nucleotide that donates its O2′ to A2b is immediately 3′ to c1.

We have found in standard k-turns that the hydrogen bond donated by the L1 O2′ is particularly important for ion-induced folding, so that folding of an L1 O2′H variant is very impaired (Liu and Lilley 2007). We made the corresponding L1 O2′H atomic variants for the *Arabidopsis* and *E. coli* k-junctions and repeated the Mg^2+^ ion titrations by measuring FRET efficiency (Fig. 2B,C). Neither k-turn variant folds well in response to the addition of Mg^2+^ ions, reaching *E*_FRET_ values <0.4, and with [Mg^2+^]_1/2_ = 1.4 and 1.0 mM, respectively (Table 1). This demonstrates the importance of the cross-strand hydrogen bond to A1n, and thus supports the assignment of the L1 position.

We also investigated the importance of the −1n O2′ in the *Arabidopsis* k-junction. We made a −1n O2′H atomic variant for that k-junction and repeated the Mg^2+^ ion titrations by measuring FRET efficiency (Supplemental Fig. S2). The ion-induced folding was impaired, though to a lesser degree than for the L1 O2′H variant. *E*_FRET_ reached 0.48, and [Mg^2+^]_1/2_ = 1.2 mM (Table 1).

### Ion-induced folding of putative k-junction in DUF-3268 RNA

While the *Arabidopsis* and *E. coli* k-junctions were first identified by their conformations within crystallographic structures of TPP riboswitches (Wang et al. 2014), DUF-3268 was proposed to be a probable k-junction on the basis of its sequence (Huang and Lilley 2018; Huang et al. 2021) within one of 224 intergenic sequences found by bioinformatic analysis (Weinberg et al. 2017). No investigation of the folding properties of this putative k-junction has been made to date. We have, therefore, made a construct based on DUF-3268 by extension of the C and NC arms, replacement of the T-helix by a 4 bp stem–loop and attachment of fluorescein and cyanine-3 fluorophores to the 5′ termini (Supplemental Fig. S1) analogously to the riboswitch k-junctions above.

**FIGURE 3.**
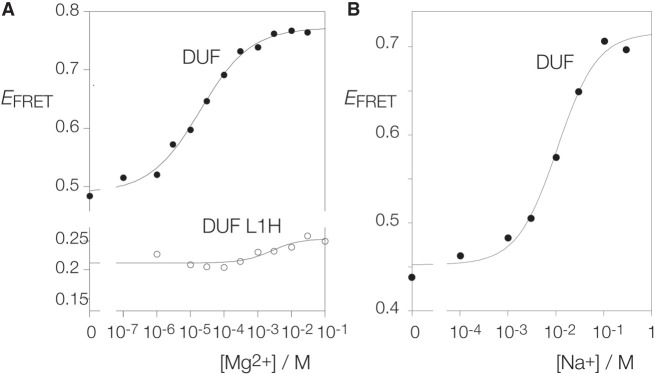
Metal ion-induced folding of the DUF-3268 k-junction analyzed by FRET. A construct analogous to that shown schematically in Figure 2A based on the DUF-3268 k-junction was used in these experiments. (*A*) Titration of the DUF-3268 k-junction with Mg^2+^ ions. *Filled circles*: natural sequence DUF-3268 k-junction. *Open circles*: DUF-3268 k-junction with L1H substitution. (*B*) Titration of the DUF-3268 k-junction with Na^+^ ions.

The addition of Mg^2+^ or Na^+^ ions to the DUF-3268 k-junction leads to an increase in FRET efficiency (Fig. 3A,B). The k-junction clearly undergoes folding into a kinked geometry in response to the addition of both divalent and monovalent metal ions, but the characteristics are significantly different from those of the *Arabidopsis* and *E. coli* k-junctions. The DUF-3268 k-junction starts from a higher value of *E*_FRET_ = 0.48 and rises to *E*_FRET_ = 0.77 (Table 1). Moreover, folding occurs in response to an order of magnitude lower concentration of both metal ions, with [Mg^2+^]_1/2_ = 20 µM and [Na^+^]_1/2_ = 11 mM (Table 1). No significant folding was observed on the addition of *A. fulgidus* L7Ae protein, with *E*_FRET_ = 0.53 in the presence of 1 µM L7Ae.

We also tested the effect of atomic mutation of the putative L1 O2′ of the DUF-3268 k-junction. Titration of Mg^2+^ ions into the L1 O2′H variant resulted in a lower initial value of *E*_FRET_ = 0.22, and a very small extent of folding (final *E*_FRET_ ≤ 0.25) (Fig. 3A). This confirms the importance of the L1 O2′ experimentally, and supports its functional assignment as L1 in the DUF-3268 k-junction structure.

### A crystal structure of the DUF-3268 k-junction

We synthesized a single 46 nt RNA oligonucleotide containing a DUF-3268 k-junction, with both the NC- and T-helices terminated by GAAA tetraloops (Supplemental Fig. S3). This crystallized in space group C121, and diffracted to a resolution of 1.94 Å. The structure was obtained by molecular replacement using three separate models, corresponding to two 5 bp stem–loops and a 5 bp helix corresponding to the NC-, T-, and C-helices, respectively, as the search model. The nucleotides of the k-junction in the crystal have been numbered sequentially in the PDB file (ID 8ITS). Crystallographic statistics are tabulated in Supplemental Table S1, and the correspondence between the numbering in the PDB file and our nomenclature for the k-junction (Liu and Lilley 2007) is shown in Supplemental Figure S4.

**FIGURE 4.**
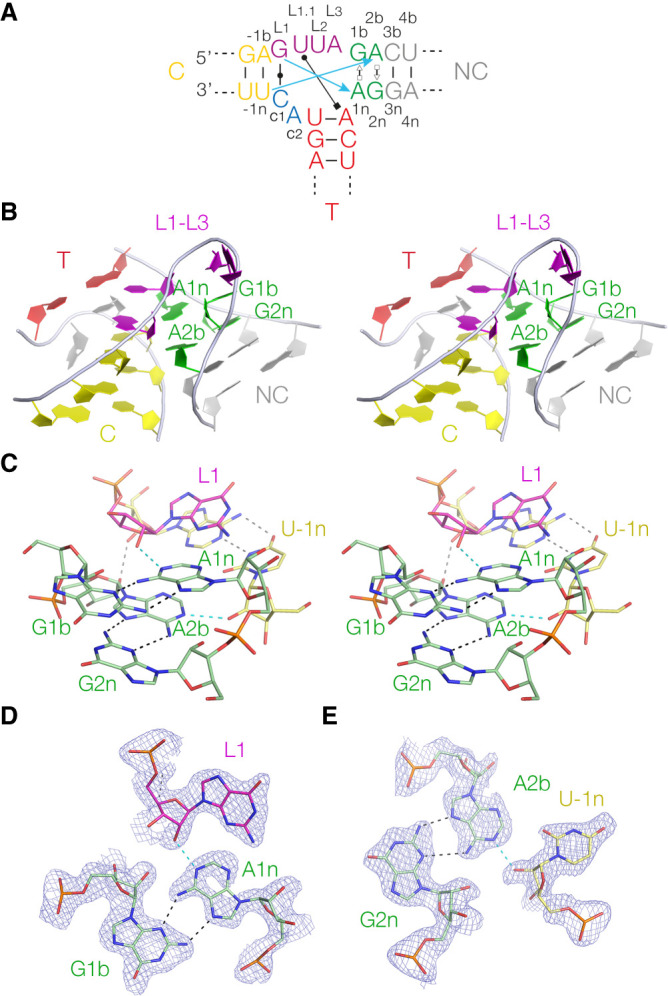
Crystal structure of the DUF-3268 k-junction. (*A*) Schematic of the DUF-3268 k-junction with long-range hydrogen bonds indicated. The blue arrows show the two cross-strand hydrogen bonds from L1 O2′ to A1n N1 and L-1n to A2b N1. Also shown is the cis Watson–Crick c1:GL1, and the base pair between L1.1 and the A of the first base pair of the T-helix, so forming a triple base interaction. (*B*) Parallel-eye stereoscopic view of the k-junction. This is viewed down onto the minor groove of the L1–L3 loop. In this view, the T-helix lies at the back. (*C*) Parallel-eye stereoscopic view of the core of the k-junction. The two sheared G:A base pairs are clearly visible, and the cross-strand L1 O2′ to A1n N1 and L-1n to A2b N1 hydrogen bonds are highlighted in cyan. (*D*) The G1b:A1n cis sugar:Hoogsteen base pair with the L1 O2′ to A1n N1 hydrogen bond (cyan). The *F*_o_–*F*_c_ electron-density map is show contoured at 1σ. (*E*) The G2n:A2b cis sugar:Hoogsteen base pair with the U-1n O2′ to A2b N1 hydrogen bond (cyan). The *F*_o_–*F*_c_ electron-density map is shown contoured at 1σ.

The overall architecture of the DUF-3268 k-junction is very similar to that of the *Arabidopsis* and *E. coli* k-junctions, forming a three-way helical junction in which the C- and T-helices are coaxial, and the axis of the NC-helix includes an acute angle with that of the C-helix (Supplemental Fig. S5). The structure of the DUF-3268 k-junction is compared with those of *Arabidopsis* and *E. coli* plus the Kt-7 k-turn are shown in Supplemental Figure S6. They superimpose well, except for a reorientation of the T-helix in DUF-3268 compared to the other k-junctions. Figure 4A shows a schematic of the structure, and the structure of the core of the k-junction is shown in Figure 4B and C. The two noncanonical sheared base pairs G1b:A1n and A2b:G2n are both *trans* G(sugar):A(Hoogsteen), as in standard k-turns. These participate in the standard cross-strand hydrogen bonds (Fig. 4C–E). As predicted, the O2′ of the ribose designated L1 donates a hydrogen bond to A1n N1 (Fig. 4C,D). Similarly, the O2′ of the ribose assigned as −1n donates a hydrogen bond to A2b N1 (Fig. 4C,E). These are fully consistent with the folding data on the atomic variants shown above and the crystallographic structure confirms their role in the k-junction. The RNA conformation corresponds to that of an N1-type k-turn, that is, A2b accepts a proton at N1. The *Arabidopsis* and *E. coli* k-junctions similarly adopt the N1 conformation in the TPP riboswitches (Wang et al. 2014).

The nucleobases of the loop (principally L1, L1.1, and L2) lie on the major groove side of the junction. The nucleobases are stacked in a manner equivalent to a standard k-turn, so that L1 is stacked on the C-helix, L1.1 is stacked on L1, L2 is stacked on the NC-helix, while L3 is directed away from the RNA. The first connecting nucleotide c1 forms a *cis* Watson–Crick C:G base pair with L1 (Supplemental Fig. S7A). Equivalent base pairs are made in the *Arabidopsis* and *E. coli* k-junctions. However, the interaction made by the second connecting nucleotide c2 adopts a different conformation from that of the riboswitch k-junctions. Instead of base pairing with L1.1 it is extended out, and UL1.1 forms an in-plane triple base interaction with the major groove edge of the junction-proximal base pair of the T-helix (Supplemental Fig. S7B). The Watson–Crick edge of the nucleobase of UL1.1 forms two hydrogen bonds to the Hoogsteen edge of the adenine, UL1.1 N3 to A N7 and A N6 to UL1.1 O4. Lastly, the structure is buttressed by two ribose–ribose interactions where the backbones approach relatively closely. One is between the O2′ atoms of A1n and c1, and the other between the O2′ atoms of A2b and A-1b. Thus, in the DUF-3268 k-junction structure, both adenine nucleotides of the sheared G:A base pairs make two cross-strand interactions using the extreme ends of the nucleotide.

### Effect of the bulged adenine on k-junction folding

**FIGURE 5.**
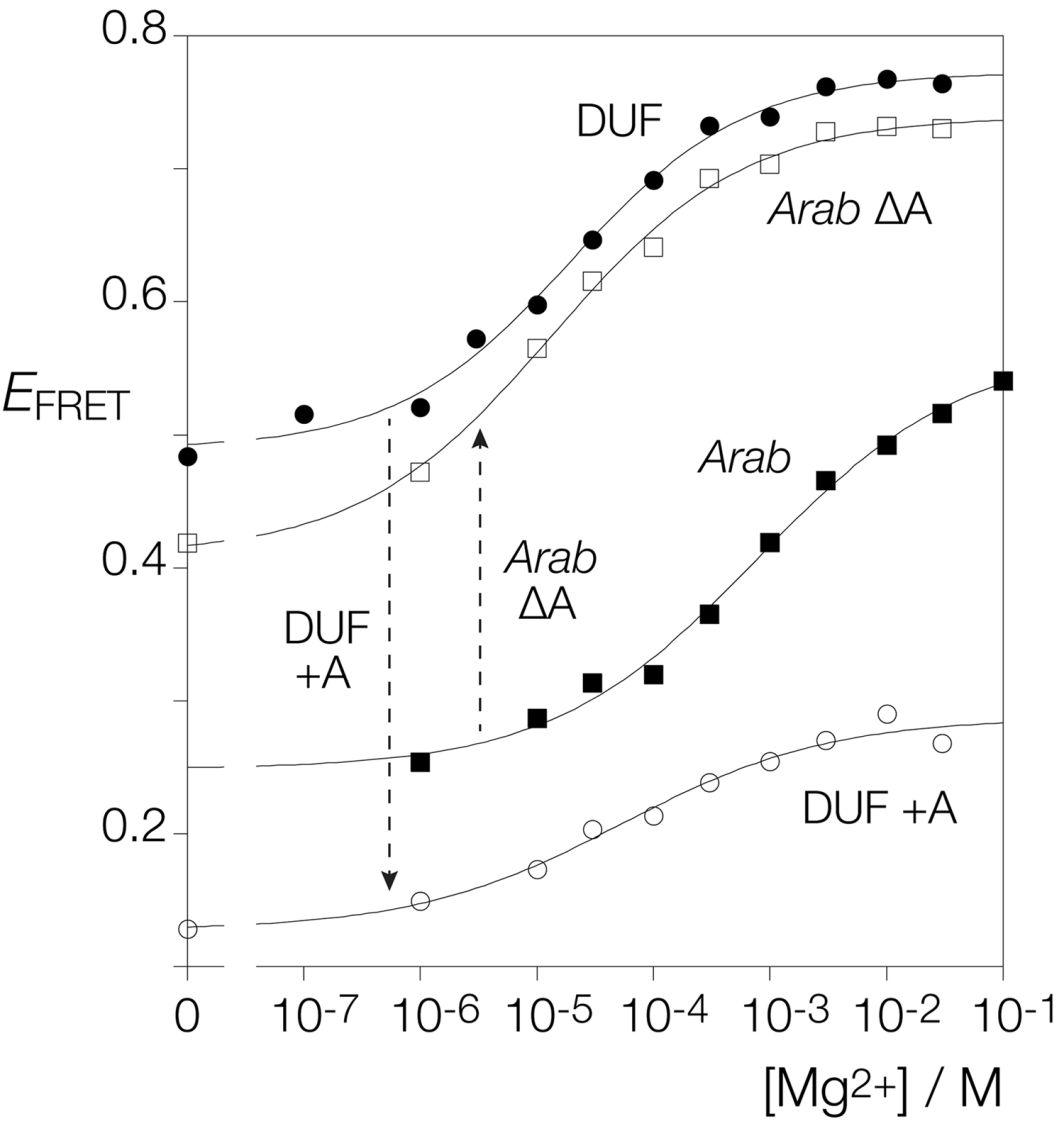
The effect of a bulged adenine inserted between G1b and A2n on the folding of the *Arabidopsis* and DUF-3268 k-junctions. The fluorescent constructs of the *Arabidopsis* and DUF-3268 k-junctions were modified so that the bulged adenine was deleted from the former, and an adenine was inserted between G1b and A2n in the latter. All four species were titrated with Mg^2+^ ions. *E*_FRET_ has been plotted against Mg^2+^ ion concentration for the *Arabidopsis* k-junction unmodified (*Arab*; filled squares) and with the bulged A deleted (*Arab* ΔA; open squares), and for the DUF-3268 k-junction unmodified (DUF; filled circles) and with the inserted adenine (DUF +A; open circles). The data are fitted to a simple two-state binding model (lines).

The most significant difference between the DUF-3268 k-junction and those of *Arabidopsis* and *E. coli* is the bulged nucleotide(s) between G1b and A2b. This is a single adenine in the *Arabidopsis* k-junction and the dinucleotide UC in that of *E. coli*. In the DUF-3268 k-junction there is no nucleotide inserted between G1b and A2b. We asked the question whether or not the difference between the folding characteristics of the *Arabidopsis* and DUF-3268 k-junctions might arise from the bulged A of the former. We therefore made a version of the *Arabidopsis* k-junction lacking the A-bulge, and a version of the DUF-3268 k-junction in which an A was inserted between G1b and A2b. These were analyzed by measuring FRET efficiency as a function of Mg^2+^ ion concentration as previously (Fig. 5).

Removal of the A-bulge from the *Arabidopsis* k-junction led to an Mg^2+^ ion titration curve closely similar to that of the DUF-3268 k-junction. The initial *E*_FRET_ = 0.42, and the maximum value achieved was *E*_FRET_ = 0.73 (Table 1). In addition, the [Mg^2+^]_1/2_ = 13 µM. In contrast, the creation of an A-bulge between G1b and A2b in the DUF-3268 k-junction severely impaired Mg^2+^-induced folding. The value of FRET efficiency in the absence of metal ions was lowered, and the maximum value was only *E*_FRET_ = 0.29, with a [Mg^2+^]_1/2_ = 53 µM. Thus, for both the *Arabidopsis* and DUF-3268 k-junctions, we conclude that ion-induced folding is impaired when there is a bulge between G1b and A2b, and significantly improved when absent.

### Functional substitution of the DUF3268 k-junction in the *E. coli* TPP riboswitch

**FIGURE 6.**
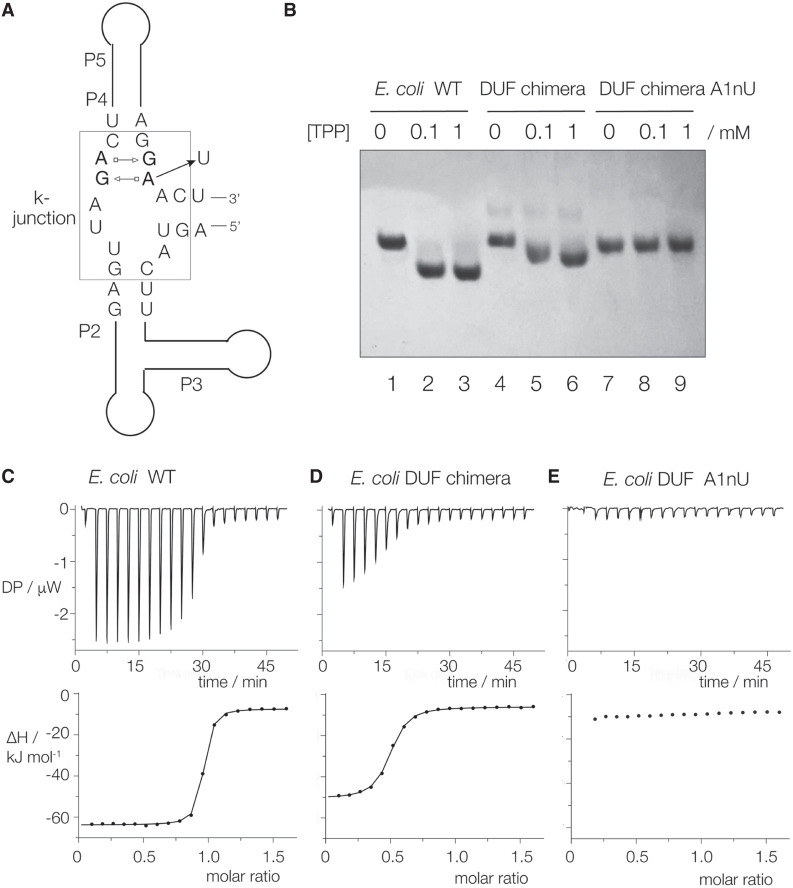
The natural k-junction can be functionally substituted by the DUF-3268 k-junction sequence in the *E. coli* TPP riboswitch. (*A*) Schematic showing a chimeric TPP riboswitch with the DUF-3268 k-junction (boxed). The position of the A1nU substitution is shown. (*B*) Analysis of the ligand-induced global conformation of the TPP riboswitch variants by polyacrylamide gel electrophoresis. The natural sequence *E. coli* TPP riboswitch and the DUF-3268 chimera with and without the A1nU substitution were electrophoresed in a 10% polyacrylamide gel after incubation with no ligand, 0.1 or 1 mM TPP. (*C*–*E*) ITC of the three riboswitch variants. A solution of TPP was titrated into the riboswitch solutions, and the heat evolved was measured as the power required to maintain zero temperature difference with a reference cell. Integration over time gives the heat required to maintain thermal equilibrium between cells. In each case the *upper* panel shows the raw data for sequential injections of 2 µL volumes (following an initial injection of 0.4 µL) of a 250 µM solution of TPP into 200 µL of a 30 µM RNA solution in 40 mM HEPES (pH 7.5), 100 mM KCl, 10 mM MgCl_2_. This represents the differential of the total heat (i.e., enthalpy Δ*H*° under conditions of constant pressure) for each TPP concentration. The *lower* panels show the integrated heat data fitted (where possible) to a single-site binding model. The thermodynamic parameters calculated are summarized in Supplemental Table S2.

We do not know the function of the DUF-3268, but since it adopts the k-junction conformation we asked whether or not it could functionally substitute for the k-junction in the *E. coli* TPP riboswitch. We engineered the *E. coli* TPP riboswitch so that its natural k-junction was replaced by the DUF-3268 sequence with the preservation of the correct spacings (Fig. 6A; Supplemental Fig. S8). We further made an A1nU point mutation in the DUF k-junction sequence; mutation in that position is known to impair folding (Wang et al. 2014). We then asked if the addition of TPP would induce a change in conformation in the chimeric riboswitch resulting in a difference in electrophoretic mobility, and whether or not TPP binding could be detected by isothermal titration calorimetry (ITC).

The electrophoretic analysis is shown in Figure 6B. The natural *E. coli* riboswitch, the DUF chimera and the DUF A1nU chimera were electrophoresed in a 10% polyacrylamide gel following the addition of no ligand, 0.1 or 1.0 mM TPP. A clear increase in electrophoretic mobility occurs with the addition of TPP. Mobility is also increased for the DUF-3268 chimeric riboswitch, although to a smaller degree than the native *E. coli* riboswitch. However, the inclusion of the A1nU mutation into the DUF sequence completely prevented the TPP-dependent change in electrophoretic mobility.

The calorimetric data are shown in Figure 6C–E, and the resulting thermodynamic data are tabulated in Supplemental Table S2. TPP was titrated into a 30 µM solution of RNA in 40 mM HEPES (pH 7.5), 100 mM KCl, 10 mM MgCl_2_ at 25°C. The native *E. coli* riboswitch binds one molecule of TPP in an exothermic reaction, from which we calculate a dissociation constant *K*_d_ = 23.9 ± 2.6 nM (Fig. 6C). The DUF-3268 chimeric riboswitch also binds TPP, but an order of magnitude more weakly, with *K*_d_ = 248 ± 23 nM (Fig. 6D). However, no binding could be detected on titration of TPP into the DUF A1nU chimeric riboswitch (Fig. 6E).

The results from the gel electrophoresis (i.e., the RNA global conformation) and the calorimetry (affinity of ligand binding) indicate that the DUF-3268 can functionally substitute for the *E. coli* k-junction in the TPP riboswitch, but binds the ligand less well than the native sequence. This suggests that subtle conformational differences between the alternative junctions have a significant influence on ligand binding and the overall conformation of the riboswitch.

## DISCUSSION

In these studies we have shown that k-junctions fold into a tightly kinked geometry on the addition of metal ions. We have further shown that the DUF-3268 sequence that was thought likely to adopt a k-junction conformation (Huang et al. 2021) does indeed do so. And we have found that the DUF-3268 k-junction can functionally substitute for that of the *E. coli* TPP riboswitch.

The folding properties of k-junctions parallel those of k-turns. In the absence of metal ions in solution, the k-junctions adopt a relatively extended conformation such that there is a low efficiency of energy transfer between fluorophores attached to the ends of the extended C- and NC-helices. On the addition of metal ions, the k-junctions undergo a change of conformation, kinking the structure so that FRET between the fluorophores increases significantly. The binding isotherms are well fitted by a two-state model. Folding of the k-junctions is induced by the addition of either Mg^2+^ or Na^+^ ions, where the half-metal ion concentration for the monovalent ions is three orders of magnitude higher than that for the divalent ions. This strongly suggests that folding responds to the neutralization of phosphate charge by atmospheric (or diffuse) binding, not site-specific binding that involves the formation of an inner sphere complex with the metal ion (Bai et al. 2005, 2007; Draper et al. 2005; Sun and Chen 2019). Moreover, no direct coordination of metal ions was observed in the core of the k-junction in the crystal, although we have observed site-specific binding of magnesium ions in the major groove of the G:A base pairs of k-turns (McPhee et al. 2014). These folding characteristics are closely similar to those of a standard k-turn such as *H. marismortui* Kt-7 (Liu and Lilley 2007).

One respect in which k-turns and k-junctions differ is that while the L7Ae family of proteins are powerful inducers of folding of k-turns with pM affinity (Turner et al. 2005; Wang et al. 2012; Huang and Lilley 2013), no folding was promoted by the addition of *A. fulgidus* L7Ae to the *Arabidopsis* or DUF-3268 k-junctions. When bound to k-turns such as *H. marismortui* Kt-7, L7Ae binds in the expanded major groove running on the outer face of the structure from the C-helix through to the NC-helix (Vidovic et al. 2000; Hamma and Ferré-D'Amaré 2004; Moore et al. 2004; Huang and Lilley 2013). In the k-junctions, access to the C-helix side will be severely hindered by the presence of the T-helix and the base interactions made by L1 and L1.1. This would likely block the binding of L7Ae.

DUF-3268 was identified among a collection of potentially structured RNA species from intergenic sequences in bacteria (Weinberg et al. 2017), and we proposed that it was very likely to form a k-junction structure (Huang and Lilley 2018; Huang et al. 2021). We have shown here that DUF-3268 does indeed adopt the k-junction conformation in the crystal, and that it undergoes folding in solution in response to the addition of metal ions in a manner similar to k-turns and other k-junctions.

The core of the DUF-3268 k-junction comprises the two *trans* A(Hoogsteen):G(sugar) base pairs, in common with standard k-turns and the k-junctions. The two adenine nucleobases accept hydrogen bonds from the L1 and −1n 2′-hydroxyl groups, explaining their ≥99% identity conservation in the *Arabidopsis* TPP riboswitches (Wang et al. 2014). We have demonstrated that removal of the L1 O2′ from either *Arabidopsis* or DUF-3268 k-junctions substantially impairs folding in response to the addition of Mg^2+^ ions. We also observed that the O2′ groups of both the A1n and A2b make further cross-strand O2′–O2′ interactions to stabilize the folded structure.

The folding characteristics of the k-junctions of the newly discovered DUF-3268 sequence and the *Arabidopsis* TPP riboswitch differ significantly in two respects. Compared to the *Arabidopsis* k-junction, that of DUF-3268 folds in response to a 40-fold lower concentration of either divalent or monovalent cations (Table 1). Second, the maximum efficiency of FRET achieved on the addition of metal ions is higher for the DUF-3268 k-junction (*E*_FRET_ = 0.77) than for the *Arabidopsis* k-junction (*E*_FRET_ = 0.57) (Fig. 5). The most significant difference in sequence between the k-junctions of DUF-3268 and the *Arabidopsis* TPP riboswitch is the adenine nucleotide inserted between G1b and A2b in the latter. Removal of the bulged adenine from the *Arabidopsis* k-junction changes its folding properties to be closely similar to those of DUF-3268, both in terms of the maximum *E*_FRET_ value, and the ionic concentration required to achieve it. Conversely, insertion of an adenine between G1b and A2b of the DUF-3268 k-junction led to the impairment of ion-induced folding. Folding reflects a balance between the stability of the kinked structure and electrostatic repulsion, and the latter will be lowered by ionic screening of phosphate groups. Thus the reduced requirement for metal ions in the absence of the bulged adenine indicates that the bulge destabilizes the k-junction.

The difference in the maximum value of *E*_FRET_ with and without the adenine bulge must reflect a conformational change in the global structure of the RNA. *E*_FRET_ is related to *r*, the distance between the donor and acceptor fluorophores (fluorescein and cyanine 3, respectively, here) by (Förster 1948):

(1) $$E_{\mathrm{FRET}}=\frac{1}{1+{\left(\frac{r}{R_{0}}\right)}^{6}},$$

where *R*_0_ is the Förster length (in Å) for a given pair of fluorophores, defined by

(2) $${R_{0}}^{6}=8.785\times 10^{-5}\frac{\kappa^{2}Q_{D}}{n^{4}}J,$$

where *Q*_D_ is the fluorescent quantum yield of the donor, *n* is the refractive index of the medium, and *J* is the spectral overlap between the emission of the donor and the excitation of the acceptor. The parameter κ depends on the relative orientation of the transition moment vectors of the two fluorophores. If both fluorophores adopt a constrained position on the nucleic acid this can vary significantly (Lewis et al. 2005; Iqbal et al. 2008; Fessl and Lilley 2013; Fuchtbauer et al. 2017). However, fluorescein is freely mobile and attached via a flexible linker, and because of its negative charge it is repelled by the RNA; in this situation κ^2^ can be assumed to take an isotropic mean value of 2/3 (Dale and Eisinger 1976; Dale et al. 1979). Because of the sixth power dependence on distance, *E*_FRET_ is highly sensitive to small changes in distance, particularly in the 0.2–0.8 range as they are here (Supplemental Fig. 9). The observed change from *E*_FRET_ = 0.57 to 0.77 would correspond to a shortening of the inter-fluorophore distance of 7.5 Å (assuming *R*_0_ = 56 Å for fluorescein-cyanine 3). Using molecular graphics, we have taken the crystal structures of the *Arabidopsis* (Thore et al. 2006) and DUF-3268 k-junctions and extended the length of the C- and NC-helices by fusion of A-form RNA helices to model the forms used for FRET measurements (Supplemental Fig. 10). There is a small reduction of the angle included between the helical axes from 36° in the *Arabidopsis* k-junction to 31° in the DUF-3268 k-junction. The bulged adenine of the *Arabidopsis* k-junction also results in a local unwinding, with the rotation about the helical axis between the G1b:A1n and A2b:G2n base pairs increasing by 10° from the DUF-3268 to the *Arabidopsis* k-junction (Supplemental Fig. 11). As a result of these conformational changes, the distance between the phosphates that are the points of attachment of the fluorophores increases by 6 Å (Supplemental Fig. 10). This is not a big change in conformation, but this simple analysis of the conformational change accounts well for the observed change in *E*_FRET_ between the two k-junctions.

Currently, there is no known function of the k-junction beyond acting as an architectural element in RNA structure, found in the TPP riboswitches and the ribosome. The identification of DUF-3268 adds a further example, so that the k-junction is an architectural motif of increasing importance. In the riboswitches, the junction is not directly involved in ligand binding. Rather, the k-junction sets up the trajectory of the loop-containing helices that act together to form the binding sites for the TPP ligand. At the present time, no function has been ascribed to DUF-3268. Its k-junction can substitute for the natural k-junction of the *E. coli* TPP riboswitch, and the chimeric riboswitch can bind ligand indicating that the k-junctions can be exchanged with preservation of function to some degree. However, the *E. coli*: DUF-3268 chimeric riboswitch binds TPP an order of magnitude more weakly than the natural *E. coli* riboswitch (Supplemental Table 2), despite the established stability of the DUF-3268 k-junction. The likely reason for this lies in the differences in conformation between the k-junctions. The TPP ligand is bound between the two helices that radiate from the k-junction, held at its two ends. The pyrimidine ring and the pyrophosphate moieties of TPP bind in internal loops contained in the extensions of the C- and NC-helices, respectively, of the k-junction (Serganov et al. 2006; Thore et al. 2006). The relative orientation and spacing of these binding loops will vary as the conformation of the junction alters, analogously to the changes of fluorophore positions discussed above. Changing the relative positioning of the binding sites even by the relatively subtle alterations we have discussed above is evidently sufficient to lower the stability of the ligand–riboswitch complex.

Our results show that k-junctions have similar folding properties to those of simple k-turns, and that DUF-3268 is another example. The local and global structure of the k-junction can be perturbed by sequence differences in the core, and these can significantly affect the function a riboswitch containing the junction. It is important to study the conformational properties of these elements, and how they might be perturbed by variation in local sequence. This information will be important for the increasingly successful efforts to model RNA structure from the sequence. Furthermore, in terms of RNA design, as our understanding of RNA motifs increases, the combination of k-turns, k-junctions, and other motifs will undoubtedly lead to exciting and novel RNA structures with new functions, and help achieve more versatile RNA architecture.

## MATERIALS AND METHODS

### RNA synthesis

RNA oligonucleotides were synthesized using phosphoramidite chemistry (Beaucage and Caruthers 1981) implemented on an Applied Biosystems 394DNA/RNA synthesizer as described in Wilson et al. (2001). Ribonucleotide phosphoramidites were protected by 2′O-tert-butyldimethyl-silyl (*t*-BDMS) (Hakimelahi et al. 1981; Perreault et al. 1990) (Link Technologies). All oligoribonucleotides were redissolved in 100 µL of anhydrous DMSO and 125 µL triethylamine trihydrofluoride (Sigma-Aldrich) to remove *t*-BDMS groups, and agitated at 65°C in the dark for 2.5 h. After cooling on ice for 10 min, the RNA was precipitated with 1 mL of butanol, washed once with 70% ethanol and suspended in double-distilled water. Fluorescein (Link Technologies) and Cy3 (GE Healthcare) were attached to the 5′ termini of the oligonucleotides as phosphoramidites in the final synthesis cycle as required.

### RNA purification and hybridization of RNA for fluorescence

RNA was purified by gel electrophoresis in 16% polyacrylamide in 90 mM Tris-borate (pH 8.3), 10 mM EDTA, 7 M urea at 23 W constant power at room temperature. RNA-containing bands were excised and recovered by elution from the crushed fragments. The eluted RNA was precipitated with ethanol and dissolved in 0.1 M TEAA. Fluorescent RNA strands were purified by reversed-phase chromatography on a C_18_ column using an 0.1 M TEAA/acetonitrile gradient. Strands were hybridized in 50 mM Tris-HCl (pH 7.5), 25 mM NaCl by slowly cooling an equimolar mixture of strands from 80°C to 4°C, and purified by gel electrophoresis in 12% polyacrylamide in 45 mM Tris-borate (pH 8.3), 25 mM NaCl buffer at 4°C. The hybridized species were recovered by excising the bands and elution from the crushed fragments followed by precipitation in ethanol. The sequences are listed in full in Supplemental Figure S1.

### L7Ae expression and purification

*Archeoglobus fulgidus* L7Ae was expressed as a hexahistidine fusion protein from a modified pET-Duet1 plasmid (Novagen) (Huang et al. 2011) in *E. coli* BL21-Gold (DE3) pLysS cells (Stratagene), and purified as described previously (Huang and Lilley 2013).

### FRET analysis of k-junction folding

FRET efficiency was measured from an RNA k-junction species terminally 5′-labeled with fluorescein on the bulged strand and Cy3 on the nonbulged strand (Fig. 2A). Fluorescein-Cy3-labeled RNA samples were dissolved in 100 µL 90 mM Tris-borate (pH 8.3) and absorption spectra were measured for each RNA between 400 and 600 nm using a Thermo Evolution 201 UV–visible spectrophotometer. Spectra were deconvoluted using a corresponding RNA species singly labeled with Cy3, and fluorophore absorption ratios required for FRET analysis were calculated using a program implemented in MATLAB. Fluorescence spectra were recorded in 90 mM Tris-borate (pH 8.3) at 4°C using an SLM-Aminco 8100 fluorimeter. Spectra were corrected for lamp fluctuations and instrumental variations, and polarization artifacts were removed by setting excitation and emission polarizers crossed at 54.7°. Values of FRET efficiency (*E*_FRET_) were measured using the acceptor normalization method (Clegg 1992) implemented in MATLAB. *E*_FRET_ as a function of metal ion concentration was analyzed using a model in which the fraction of folded molecules corresponds to a simple two-state model for ion-induced folding, that is

(3) $$E_{\mathrm{FRET}}=E_{0}+\Delta E_{\mathrm{FRET}}\cdot K_{A}{\left[M\right]}^{n}/(1+K_{A}{\left[M\right]}^{n}),$$

where *E*_0_ is the FRET efficiency of the RNA in the absence of added metal ions, Δ*E*_FRET_ is the increase in FRET efficiency at saturating metal ion concentration, [M] is the prevailing metal ion concentration (either Mg^2+^ or Na^+^), *n* is the Hill coefficient and *K*_A_ is the apparent association constant for metal ion binding. Data were fitted by nonlinear regression in Kaleidagraph (Synergy Software). Half-metal ion concentration was calculated as [M]_1/2_ = (1/*K*_A_)^1/*n*^.

### Synthesis of RNA by transcription

To facilitate crystallization, we introduced GAAA tetra-loop motifs into the variable loops of stem NC and stem T of the DUF-3268 k-junction (Supplemental Fig. 3). The DNA sequences were amplified by PCR to generate DNA templates. In vitro transcription was performed with T7 RNA polymerase at 37°C for 6 h, followed by purification by electrophoresis in 10% polyacrylamide gel electrophoresis under denaturing conditions. The RNA transcript was visualized by UV-shadowing at a wavelength of 365 nm, the bands excised and soaked in 0.25× TBE buffer. The eluted RNA was precipitated with isopropanol, washed with 70% ethanol, and the air-dried RNA was dissolved in double-distilled water.

### Crystallization, structure determination, and refinement

Crystals were obtained with the 46 nt RNA (Supplemental Fig. 3). The RNA was diluted to a concentration of 0.6 mM and annealed at 95°C for 1 min in a buffer containing 5 mM HEPES (pH 7.0), 100 mM KCl, 5 mM MgCl_2_ before cooling at room temperature for 10 min. An amount of 0.2 µL RNA samples were mixed with 0.2 µL reservoir solution for crystallization screening using sitting drop vapor diffusion at 18°C. Crystals of RNA diffracting to high resolution grew after 4 d from the condition 0.1 M Mg acetate, 0.05 M MES (pH 6.0), 30% v/v 2-methyl-2,4-pentanediol and 0.05 M NaF. The crystals were mounted in nylon loops and flash-frozen by plunging into liquid nitrogen. X-ray diffraction data were collected at beamline BL19U1 at the National Facility for Protein Science in Shanghai (NFPS).

Structures at 1.94 Å resolution were determined by molecular replacement using PHASER with three models; these were 5 bp stem–loops corresponding to the NC and T stem–loops, and a 5 bp helix corresponding to the C-helix with sequences as present in the complex crystallized. All the models were produced by the fully automated RNA structure modeling server RNAComposer (https://rnacomposer.cs.put.poznan.pl/). The translation function Z score (TFZ) was 12.9 and log-likelihood gain (LLG) was 289.4. The model was further adjusted using Coot and subjected to several rounds of adjustment and optimization using Coot, phenix.refine, and PDB_REDO. Model geometry and the fit to the electron-density maps were monitored with MOLPROBITY and the validation tools in Coot. Simulated annealing omit maps were calculated by Composite omit map in PHENIX suite using the method anneal.

### Electrophoretic analysis of TPP riboswitch species

The *E. coli* TPP riboswitch, DUF chimeric riboswitch and its A1nU variant were analyzed by electrophoresis in 10% polyacrylamide gels in 25 mM Tris, 192 mM glycine (pH 8.3), 1 mM MgCl_2_ following preincubation in the same buffer plus 0, 100 µM, or 1 mM TPP for 5 min. at 20°C followed by the addition of 5% glycerol and 0.025% (w/v) bromophenol blue and xylene cyanol FF. Electrophoresis was performed at 4 W for 2 h, during which the temperature remained below 20°C. After electrophoresis, RNA was visualized by UV-shadowing.

### Isothermal titration calorimetry of ligand binding to TPP riboswitch species

Isothermal titration calorimetry experiments were performed on a Microcal PEAQ-ITC microcalorimeter at Sun Yat-sen University Cancer Center. Wild-type and variant *E. coli* TPP riboswitch species were dissolved to a final concentration of 30 µM in a buffer containing 40 mM Hepes (pH 7.5), 100 mM KCl, 10 mM MgCl_2_ followed by incubation on ice. TPP was dissolved in the same buffer and diluted to a concentration of 250 µM before titration into the RNA in the sample cell. Titration experiments were performed at 25°C, starting with an injection of 0.4 µL, followed by 18 consecutive injections of 2 µL TPP with 120 s intervals between injections, and a reference power of 41.9 µW. Titration data were integrated and analyzed using MicroCal PEAQ-ITC analysis software. The apparent dissociation constant (*K*_d_) was calculated from a “one set of sites” binding model. All binding constants and thermodynamic values are listed in Supplemental Table 2.

## DATA DEPOSITION

The coordinates of the DUF-3268 k-junction have been deposited with the PDB with ID 8ITS.

## SUPPLEMENTAL MATERIAL

Supplemental material is available for this article.

## Figures and Tables

**Mengxiao Li:**
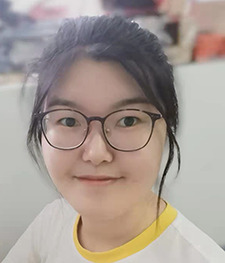


## MEET THE FIRST AUTHOR

***Meet the First Author(s)* is an editorial feature within *RNA*, in which the first author(s) of research-based papers in each issue have the opportunity to introduce themselves and their work to readers of *RNA* and the RNA research community. Mengxiao Li is the first author of this paper, “Structure and ion-dependent folding of k-junctions.” Mengxiao received her master's degree under the guidance of Dr. Yu Guo in the School of Pharmacy at Nankai University, and then joined Dr. Lin Huang's laboratory of Sun Yat-sen Memorial Hospital as a research assistant.**

**What are the major results described in your paper and how do they impact this branch of the field?**

We elucidate the folding of RNA structural motif named k-junction upon the addition of metal ions. Using FRET, we show evidence for ion-dependent folding of k-junctions from *Arabidopsis* and *E. coli* TPP riboswitches, supporting a kink-turn-like fold. Then we determined the crystal structure of a putative DUF-3268 k-junction, confirming that it belongs to the k-junction family as predicted. This systematic analysis and comparative study of RNA architectures improves our ability to predict RNA structure from sequence.

**What led you to study RNA or this aspect of RNA science?**

My interest in RNA research was sparked when I joined Dr. Lin Huang's laboratory. The laboratory is mainly engaged in the study of the structure and function of nucleic acids, including riboswitches, ribozymes, and aptamers. It is very interesting to solve their three-dimensional structures, revealing their mechanisms and functions at the atomic level. Seeing the microscopic world with our eyes is part of the allure of structural biology.

**During the course of these experiments, were there any surprising results or particular difficulties that altered your thinking and subsequent focus?**

Our work has shown that k-junctions are more widespread, not just restricted to TPP riboswitches and the ribosome.

**What are some of the landmark moments that provoked your interest in science or your development as a scientist?**

I've always loved life science. There is a famous structural biologist Shi Yigong in my hometown. He kindled my interest in structural biology.

**If you were able to give one piece of advice to your younger self, what would that be?**

Be bold and allow everything to happen.

**Are there specific individuals or groups who have influenced your philosophy or approach to science?**

My current laboratory supervisor, Lin Huang, advocates a critical, thoughtful and innovative scientific approach, together with active communication, and good collaboration. This benefits me a lot.
